# Bible Beauties

**Published:** 1870-11

**Authors:** 


// HALL'S

### JOURNAL OF HEALTH.

[*All Articles are written by the Editor.*]

VOL. XVII.—NOVEMBER, 1870.—NO. XI.

### BIBLE BEAUTIES.

Health is promoted, happiness increased, and life prolonged by the large contemplation of the beautiful in Nature, Art and Revelation. Natural philosophers live longer than any other class of men—clergymen than either of the other professions. The human mind everywhere takes in truth with pleasure; it feeds on what is new, and if the new is beautiful and true, it is a feast of fat things, nourishing the immortal part, and giving life to the body itself.

"And still new beauties do I see,
And still increasing light,"

is the expressed experience of one who took great delight in studying the Bible, which is an unfailing mine of thought, a well of water springing up unto everlasting life; its beauties will never be exhausted while time lasts—especially will they be "ever charming, ever new" to those who have studied the original languages, the Greek, the Hebrew, and the Latin; and it would be a delightful and profitable employment if societies could be formed, with regular times for meeting, for the purpose of comparing notes, in a conversational manner, as to individual ideas upon certain portions of Scripture—verses or words. Two or three classical scholars thus meeting, could instruct and delight a whole room full by the hour, by bringing out the literal meaning by a simple scholastic analysis of the roots, and themes, and derivations of leading words; thus, "Now is the accepted time, behold now is the day of salvation." The time is not "accepted" by many, but change the word to "accepting," as the original allows, and it gives out the idea which is more critically accurate, "now is the accepting time," or, now is the time for accepting the offer of salvation.

"Lead us not into temptation." It is here implied, that the All Merciful leads his children into temptation; but if we examine closely, it will authorize the use of another word, and we shall read with beautiful appropriateness, "Abandon" us not to temptation.

"No Scripture is of any private interpretation." This might be construed to mean that we must not think for ourselves in reading the Bible; hence, some have thought it best to keep the common people from reading that blessed book; but the clerical scholar will tell you that the original Greek permits the use of the word "separate," instead of private, and it would then read, "No Scripture is of any "separate" interpretation: that is, it must be construed in its proper connection; otherwise, suicide might find scripture authorization, for in one place it is said "Judas went out and hanged himself," and in another, "Go and do thou likewise."

"At home in the body, we are absent from the Lord." The true idea is very beautiful, that we are away from home until we get to Heaven; that "Dying is but going home." The Holy Scriptures are full of hidden beauties like these, and if it was only more in the fashion to study them at home, to read them more, to talk about them more, so as to get at the meaning which is found below the surface, as the purer gold is had by the digging deeper, this seething cauldron of human life, with its tempestuous tossings, its worrying ambitions, its ruinous rivalries, its carking cares, would become more like a placid sea, and we should feel, in reference to the sacred volume, that it was the book of books, and each man for himself would exclaim in rapturous enthusiasm :

"This little book I'd rather own
Than all the gold and gems
Which ever in monarch's coffers shone,
Or all their diadems:
Yes! were the seas one chyrsolite,
The earth one golden ball,
And diamonds to the stars of night,
This book were worth them all."

